# CellMethy: Identification of a focal concordantly methylated pattern of CpGs revealed wide differences between normal and cancer tissues

**DOI:** 10.1038/srep18037

**Published:** 2015-12-10

**Authors:** Fang Wang, Shaojun Zhang, Hongbo Liu, Yanjun Wei, Yihan Wang, Xiaole Han, Jianzhong Su, Dongwei Zhang, Baodong Xie, Yan Zhang

**Affiliations:** 1 College of Bioinformatics Science and Technology, Harbin Medical University, 150081, Harbin; 2 The 2nd Affiliated Hospital, Harbin Medical University, Harbin, 150081, China; 3 The Department of Cardiovascular Surgery, The Second Affiliated Hospital, Harbin Medical University, Harbin, 150081, China

## Abstract

DNA methylation patterns may serve as a key in determining cell phenotypes and functions. Adjacent CpG patterns may provide insight into methylation functional mechanisms. Some regions display different DNA methylation patterns between normal and cancer tissues, but the same average methylation level. Here, we developed a method (CellMethy) to infer a region in which all CpGs exhibit concordant methylation (CM) and to quantify the extent of CM in the region. Using simulation data, CellMethy showed high performance in discovering the concordant methylation patterns (AUC = 0.89). CellMethy was then applied to RRBS data including 11 normal tissues and 12 tumors. We found that the extent of CM exhibited wider differentials among tissues than did the average methylation levels from the CM regions, with 45% of CM regions occurring specifically in one tissue and mainly in tumors. Then, we identified CM regions through genome wide bisulfite sequencing (GWBS) data on breast cancer. Approximately 82% of CM regions revealed a significantly different extent of CM between cancer and normal tissues. CellMethy can accurately describe concordantly methylated regions, and the results suggest that CM might also serve as a stable marker of cell sub-populations.

Genomes of multiple species are tagged by epigenetic markers, including the methylation of cytosine within DNA. DNA methylation is one of the most important epigenetic modifications and plays important roles in germline development^1^,^2^, embryogenesis^3^, and somatic differentiation^4^,^5^,^6^. Methylation modifications throughout the genome are referred to as the ‘methylome’^7^. DNA methylation has been shown to occur in both regional and preserved local activity states, such as during gene transcription^8^. DNA methylation patterns may serve as a key in determining cell phenotypes and functions. Recently, a large number of studies have identified numerous differential regions based on average methylation levels across tissues^9^,^10^,^11^. In addition, many cancer-related hyper-methylated and hypo-methylated regions have been found^10^,^12^,^13^,^14^, and several onco- and tumor-suppressor genes frequently alter epigenetic states in tumors^15^. However, DNA methylation patterns are highly divergent among various cell types, especially comparing tumor and normal cells^16^,^17^,^18^. Unlike the genomic DNA sequence, the epigenome is variable among tissues/cells even from the same individual^19^. There are at least as many methylomes as cell types, and fluctuations occur within a single cell according to cellular and environmental conditions^20^. DNA methylation patterns within a cell population from somatic tissue are highly heterogeneous and polymorphic^21^. Currently, more high-throughput sequencing data are available, which make possible to observe each methylation pattern in cell populations.

Although average DNA methylation levels have proven their powers, the mechanism of underlying different methylation patterns remains poorly understood. Some studies have observed that adjacent CpGs within a region exhibit co-methylation states, especially within CpG islands (CGI)^22^,^23^. The methylation patterns of adjacent CpGs may provide insight into methylation functional mechanisms. Different methylation patterns within a cell population may result in an identical average methylation level of the region but represent the outcomes of markedly different epigenetic mechanisms. We have termed the adjacent concordantly methylated CpG patterns in a region focal concordantly methylated patterns.

Here, we aimed to identify concordantly methylated patterns of adjacent CpGs using high-throughput, single-base-resolution DNA methylation data. Adjacent CpGs within a region tend toward co-methylation, and the aberrance of concordant methylation between adjacent CpGs in specific regions is often invoked as a direct driver of the carcinogenic process. Therefore, we focused on the focal concordant methylation of adjacent CpGs. A computational approach (CellMethy) was developed to identify regions containing concordantly methylated DNA (CM region, CMR) and to quantify the extent of genomic regions that share a common concordant methylation status. Tthe methylation status in each sequence read, called an epiallele^21^, can be regarded as a representation of the “haplo-methy-type” in each cell. The ratio of concordant methylation “haplo-methy-type” can be estimated as a novel biomarker representing the cell sub-population. CellMethy can be used to analyze methylation patterns in mixed cell populations, including tumor cells; may be beneficial in exploring cell subpopulations with unique DNA methylation patterns and can be regarded as a biomarker representing a cell subpopulation.

## Results

### Overview of CellMethy

**Figure 1:**
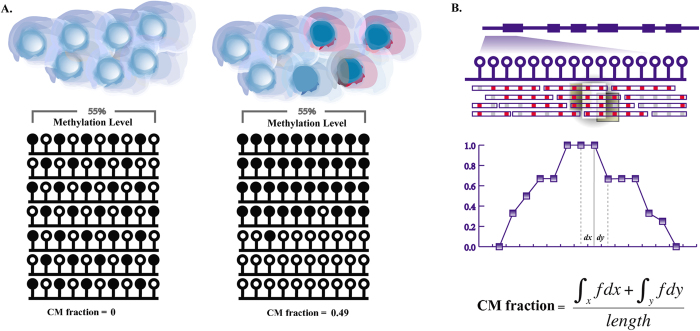
Outline of CellMethy. (**A**) Diagram of concordant methylation in cell. Balls indicated by blue shading represent individual cells from the tissue. Filled and empty circles represent methylated and unmethylated CpGs, respectively. Rows represent methylation patterns of each sequencing read. The regions in different somatic tissues showed similar average methylation levels (55%) but different methylation patterns. CM fractions represent the extent of concordant methylation of adjacent CpGs in the region. (**B**) Flowchart of identification and quantification of CMR. Empty circles represent CpGs in the human genome. Blue empty bars represent sequencing reads, in which red and gray represent methylated and unmethylated states corresponding to the genome CpG. Scatter points in the fitting curve represent the CM fraction of sliding windows, the horizontal axis represents the physical position of the genomem, and *dx* and *dy* represent the physical distance of two adjacent siliding windows.

CellMethy was developed to identify CMR and quantify its extent in a cell population based on single-base-resolution DNA methylation data. The region shown in Fig. 1A, displayed different DNA methylation patterns but the same average methylation level (0.55), upon comparing the cell populations. However, the quantization of the CMR is great enough to reflect the differential methylation patterns between cell populations (CM fraction = 0 *vs.* 0.49). Therefore, it is very important to accurately assess DNA methylation in a cell population, as different DNA methylation patterns may result in differential epigenetic regulation mechanisms, driving multiple cell phenotypes.

A brief overview of CellMethy is outlined in Fig. 1B. First, the reference genome was divided into small windows based on the number of CpGs after sequencing reads were mapped to the reference genome. Starting from the sliding window, the CellMethy algorithm claimed that all CpGs in the window were commonly covered by at least 10 sequencing reads. The fraction of reads in which all CpGs were concordantly methylated was calculated (CM fraction). Second, the hot spot was selected as the location in which the CM fraction was the highest in the neighborhood. We extended the hot spot to both sides of the window until the CM fraction equaled zero or the distance between two adjacent windows was greater than 100 bp. Lastly, CMRs were determined and quantified based on the definite integral strategy (see Methods).

### Identification and assessment of CMR in simulation data

Simulation datasets with four different coverage depths (10×, 20×, 50×, and 100×) were used to estimate parameters, including the length of the sliding window and the coverage depth. The state of each CpG site in each simulation dataset was generated randomly (random methylated data) or identically to the state of the adjacent CpG (concordantly methylated data) (see Methods). With the size of sliding windows ranging from 2 to 10 CpGs, CMRs were identified in both random and concordantly methylated simulation data. We found that the characteristics of CMRs, including the number of regions (Supplementary Figure 1), number of CpGs (Supplementary Figure 2), and CM fraction (Supplementary Figure 3), did not vary with coverage depth. However, the characteristics did vary with sliding window size. CM was significantly different between the random and concordantly methylated data when the sliding window length was greater than 4. Moreover, the probability distribution of the CM fraction was similar to the theoretical uniform distribution in random methylated data but similar to the bimodal distribution in concordantly methylated data. Thus, the sliding window length and the least coverage depth were defined as 5 and 10× in the following analysis, respectively.

**Figure 2:**
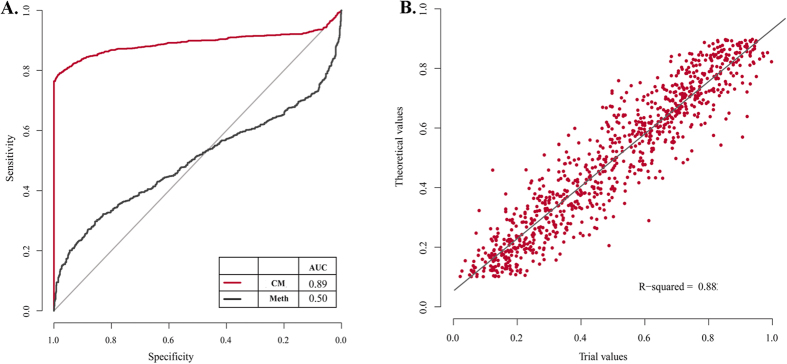
Performance evaluation on identification and quantification of CMRs based on simulation data. (**A**) Receiver operating characteristic curve (ROC) of CMR. Red lines represent the ROC curve of CellMethy with the AUC value of 0.89 (CM). Black lines represent the ROC curve of average methylation levels with the AUC value of 0.50 (Meth). (**B**) The correlation between predicted and theoretical values of CM fractions. R-square was calculated by linear regression model.

The power of CellMethy in identifying concordant methylation patterns compared to average methylation levels was measured through simulation data. A methylation value randomly selected from 0.1 to 0.9 was considered the theoretical value of each region. CM (positive) and random methylation (negative) were simulated based on the theoretical value, and replication was randomized 1000 times. Both the CM fraction and the average methylation levels were estimated in each region. The theoretical value of each region in the positive set was regarded as the true CM fraction. As shown in Fig. 2A, the area under the receiver operating characteristic curve (AUC) of CellMethy was 0.89, which can accurately distinguish between concordant and random methylation patterns. When average methylation levels were used as the distinguishing indicator, the AUC value was 0.50, corresponding to the power of random prediction. Moreover, the predicted values of the CM fraction were highly correlated with the true value (R^2^ = 0.88, Fig. 2B). Above all, CellMethy not only showed high performance in distinguishing the concordant methylation pattern, but also accurately estimated the extent of CM in a cell population.

### Concordantly methylated patterns are characteristic across cells/tissues

**Figure 3:**
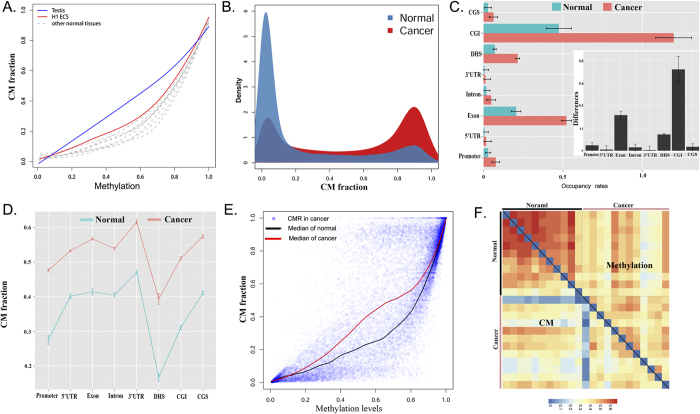
Characteristics of CM. (**A**) The relationship of CM fractions and average methylation levels in normal tissues is shown in Table 1. The maximum CM and CM of random methylation trends were computed using simulated data of concordant and random methylation pattern (methylation levels from 0.1 to 0.9), respectively. (**B**) Probability density distribution of average CM fraction from cancer and normal tissues, respectively. (**C**) Occupancy rates of CMRs in promoter, 5′-UTR, exon, intron, 3′-UTR, DHS, CGI and CGS. Box figure represents the degree of difference in occupancy rate between cancer and normal cells in each region. Notably, the occupancy rate of CGI in cancer was more than 1 because the length of some CMRs in CGI was longer than the CGI. (**D**) Average values of CM fraction of cancer and normal cells in promoter, 5′-UTR, exon, intron, 3′-UTR, DHS, CGI and CGS. (**E**) The relationship between CM fractions and average methylation levels in normal and cancer cells, respectively. (**F**) Heat map of correlation of CM fraction or average methylation levels among tissues.

We applied CellMethy to RRBS data downloaded from the Encode Project including 11 normal cells/tissues and 12 tumors. The lengths of CMRs identified in normal cells/tissues were similar, especially H1 ESC, which showed the highest CM extent among normal cells/tissues (Table 1). We found that the H1 ESC and testis showed an increased CM fraction compared to other normal tissues, corresponding to different average methylation levels, especially in the testis, which was almost linearly correlated with average methylation levels (Fig. 3A). It has been suggested that methylation patterns within germline and pluripotent cell populations maintain a stable state but undergo stochastic variation processes during subsequent somatic development. Therefore, decreased CM fraction at similar average methylation levels were observed in other somatic cells/tissues. This result was consistent with the conclusion of epipolymorphism, which was lower H1 ESC and testis^21^.

Compared to normal cells, tumor cells contained longer CMRs, involved more CpGs, and showed a higher CM extent (Fig. 3B). Moreover, the promoter, 5′-UTR, exon, intron, 3′-UTR, DNase I hypersensitive sites (DHS), CGI, and CpG island shore (CGS) all revealed higher occupancy rates of CMRs in cancer than in normal cells/tissues. The greatest difference between cancerous and normal cells/tissues was observed in CGI (Fig. 3C). Some CMRs were located in DHS and had the lowest CM fraction (Fig. 3D). Due to the inhibition of transcription from DNA methylation, regions marking active chromatin and controlling active transcription, such as the promoter and DHS, showed an inverse correlation with CM. However, smaller occupancy rates but a higher extent of CMRs were located in the CGS compared to the CGI. The CGI shore was associated with the differentiation of tissues but had lower CpG density than the CGI. It is implied that a high CM extent located in the CGI shore may be due to differences among tissues.

Combined with average methylation levels, we found that the extent of CM was significantly different between normal and cancerous cells, especially in moderately methylated regions (0.2 ~ 0.8) (Fig. 3E, Supplementary Figure 4). It is suggested that, compared to normal tissue, adjacent CpGs in moderately methylated regions are more prone to co-methylation in cancerous tissue. However, lower correlations of CM fractions were observed among cancers than in normal cells/tissues. Moreover, the correlations of CM fractions among cells were lower than the correlations of average methylation levels (Fig. 3F). Interestingly, breast cell lines (MCF) showed global differences in CMRs compared to both normal and cancer tissues. This result hinted thatthe focal concordant methylation may diverge more among tissues than their average methylation levels, and may be regarded as biomarkers of different tissues, especially in cancers.

**Figure 4:**
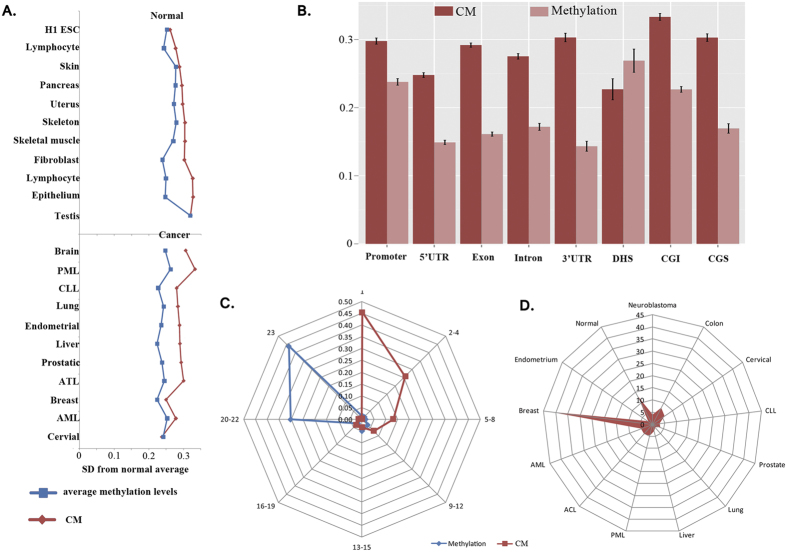
Divergence and specificity of CM. (**A**) Standard deviations of each tissue from average value of all normal tissues/cells. Blue represents the standard deviations of methylation levels. Red represents the standard deviations of CM fractions. (**B**) Differential degree between normal and cancer cells in promoter, 5′-UTR, exon, intron, 3′-UTR, DHS, CGI and CGS regions. Dark red represents the differential degree of CM fractions, while light red represents the differential degree of methylation levels. (**C**) Distribution of the number of samples that shared the same CM or methylation regions. Blue represents CMRs with methylation levels greater than 0, and red represents a CM fraction greater than 0. (**D**) Distribution of CMRs (%) among somatic tissues. Normal represents all normal tissues/cells; others represent each cancer.

Thus, the average quantity of CM in normal tissue and the standard deviation within each tissue were calculated. As expected, CM showed greater variation than the average methylation levels both in normal and cancerous tissues, with the greatest variation observed in cancer (Fig. 4A). Moreover, the average differential degrees of the CM fraction between cancerous and normal tissue within the promoter, 5′-UTR, exon, intron, 3′-UTR, CGI, and CGS were greater than the average methylation levels (Fig. 4B). Distribution of the number of samples that shared the same CM or methylation regions revealed that although the vast majority (approximately ~45%) of CMRs were methylated in all 23 tissues, more than 45% of CMRs revealed a concordantly methylated pattern only in one tissue and were enriched primarily in cancer cells (Fig. 4C), suggesting concordant methylarion is highly specific. It is noteworthy that these cancer-specific CMRs were primarily enriched in breast cancer (Fig. 4D). These results indicate that the extent of concordant methylation exhibits greater differences among cells/tissues and has specificity in cancerous cells. This result suggests that concordant methylation is more likely to be a characteristic of the cancer methylome.

### Widespread differences in focal concordant methylation between breast cancer and normal tissue

CellMethy was also successfully applied to a GWBS dataset including one HCC1954 breast cancer cell line (HCC) and one normal primary human mammary epithelial cell line (HMEC) and identified 1723 CMRs in total. There were 1093 and 835 CMRs identified in HCC and HMEC, respectively (Supplementary Table 1). The number of CMRs in HMEC accounted for less than half of the total CMRs, and the overlaps between cancer and normal tissue were less. The median value of the CM fraction was 0.38 in HCC and 0 in HMEC from all 1723 CMRs, displaying a more significant difference than their average methylation levels (Supplementary Figure 5).

**Figure 5:**
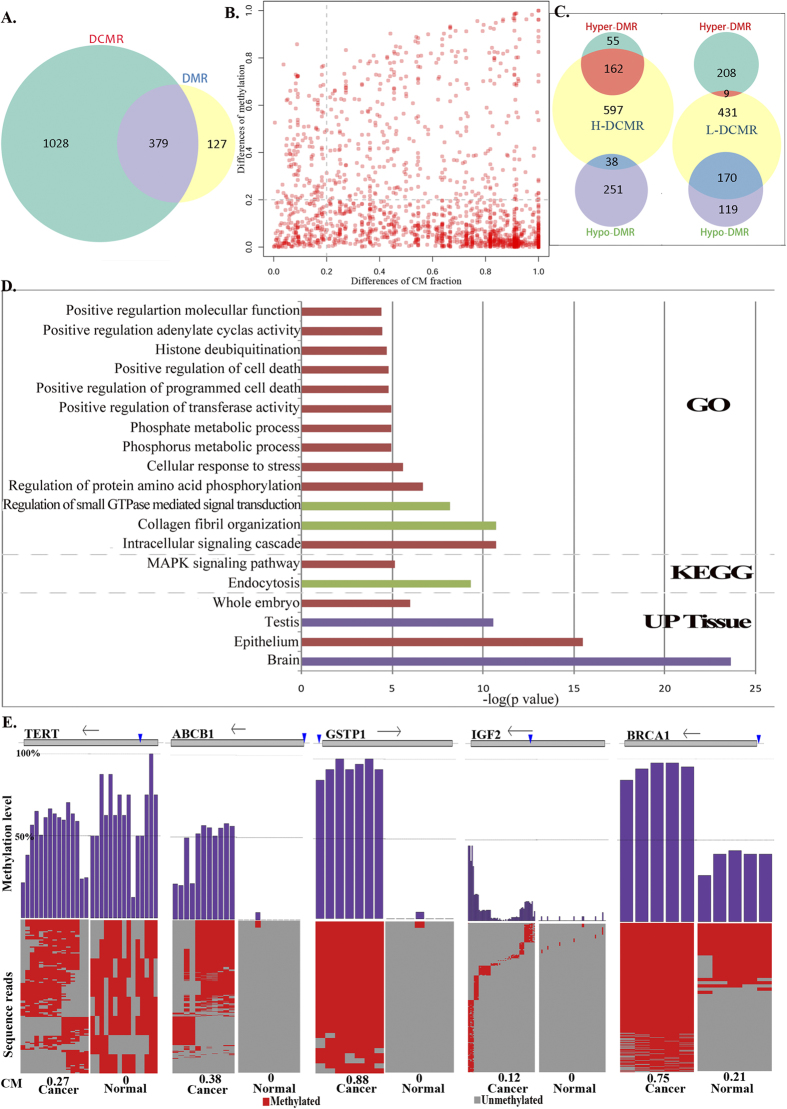
DCMRs of breast cancer from GWBS data. (**A**) DCMRs and DMRs. (**B**) Differential degree of CM fractions and average methylation levels in CMRs. The dotted line represents the cutoff of variance. (**C**) Overlap between differential CM and methylation regions. H(L)-DCMR represents higher (lower) CM fractions in cancer than normal cells, and Hyper(Hypo)-DMR represents higher (lower) average methylation levels in cancer than normal cells. (**D**) Enrichment analysis of DCMRs that did not show differential methylation levels. Red represents functions enriched in H-DCMRs, green represents functions enriched in L-DCMRs, and purple represents functions enriched in both H-DCMRs and L-DCMRs. (**E**) An example of CM patterns from TERT, ABCB1, GSTP1, IGF2 and BRCA1 that were associated with breast cancer in previous studies. The gray rectangle corresponding to each gene represents the genomic position. Vertical lines represents the locations of CMRs, and arrows represents the transcriptional direction of each gene. Purple histograms represents the average methylation level of each CpG in CMR from sequencing reads. Heat map shows methylation state of each CpG in sequencing reads in HCC1954 (left, cancer) and HMEC (right, normal). Red is methylated and gray is unmethylated.

Differential methylation region (DMR) and differential CM region (DCMR) were identified respectively through the same criterion which were at least 0.2 differences in average methylation level or CM level. We further identified 1407 DCMRs in HCC, most of which revealed a greater CM fraction in the cancerous cells. On the other hand, 506 DMRs were identified with an absolute difference in average methylation levels of more than 0.2. Most DMRs revealed hypo-methylation in cancer, which is distinct from differential CMRs (DCMRs). As shown in Fig. 5A, approximately 27% of the differential CMRs overlapped with 75% of the DMRs. A large number of differential CMRs did not overlap with DMRs, but they showed a significant difference between HCC and HMEC (Fig. 5B). These results illustrate that the extent of focal concordant methylation is more distinct between breast cancer and normal cells than their average methylation levels. In addition, we found some regions with decreased average methylation levels in cancer but increased CM fractions. The number of these regions was higher than in cancerous cells whose average methylation levels increased, but whose CM fractions decreased (38 *vs.* 9, Fig. 5C). It is suggested that adjacent CpGs prefer concordant methylation in tumors. Functional enrichment analysis of DCMRs revealed that multiple Gene Ontology (GO) functional terms were significantly enriched. Those regions exhibiting an increased CM fraction in cancer are associated with more functions, such as molecular function regulation, cell death regulation, phosphate metabolic processes, and intracellular signaling cascades. In addition, regions with an increased CM fraction in cancer were significantly associated with the MAPK signaling pathway and were up-regulated in normal epithelial cells (Fig. 5D). Together, the extent of concordant methylation was larger in tumors than in normal tissue and seems to reflect a dynamic mechanism of methylation that drive the formation of tumor cells.

Further analysis of genes associated with breast cancer, including ABCB1, BRCA1, GSTP1, IGF2, and TERT, showed a high CM fraction in cancer but non-CM in normal cell lines (Fig. 5E). ABCB1, BRCA1, GSTP1, and IGF2 displayed both an increased CM fraction and hyper-methylation in cancer. It is interesting that TERT exhibited higher CM in cancer than normal tissue (0.27 *vs.* 0) but a lower average methylation level (0.53 *vs.* 0.65). TERT, normally repressed in postnatal somatic cells, plays a role in cellular senescence by the progressive shortening of telomeres, and its decreased expression in somatic cells may play a role in oncogenesis. Consistent with this result, the expression of TERT is suppressed in breast cancer, as assessed by quantitative polymerase chain reaction (q-PCR)^24^, but no studies have shown variations of TERT DNA methylation in breast cancer. In our study, we found that a CGI within TERT showed a significantly differential CM fraction between cancerous and normal cells (absolute difference = 0.27), significantly higher than the difference of average methylation levels (absolute difference = 0.12) that was not identified as a DMR based on the absolute difference cutoff (0.2). Although TERT showed a higher average methylation level in normal, adjacent CpGs of the CGI within TERT preferentially showed concordant methylation in cancer cells but a random methylated pattern in normal cells. Variation in the concordant methylation pattern rather than average methylation levels of TERT may lead to deregulation of expression. Thus, we propose that focal DNA concordant methylation can more accurately reflect phenotype regulation than average methylation levels, which may drive the variation in cell phenotypes.

## Discussion

In this study, bisulfite sequencing data (BS-Seq) have been reanalyzed at the read level instead of by average methylation. We developed a method (CellMethy) to systematically identify the region in which adjacent CpGs are concordantly methylated and to quantify the extent of concordant methylation. Through CellMethy, we have analyzed different methylation datasets and found distinct methylation patterns across cancers. The cancer methylome generally exhibits a larger extent of concordantly methylated pattern than the normal methylome. Moreover, the CM extent showed greater variability than the average methylation levels among tissues/cells. In particular, approximately half of CMRs were specific to a single tissue/cell, especially cancerous ones. Of course, this finding is only a preliminary insight from our observation that needs to be studied in more cancerous and normal methylomes. In addition, we identified DMRs and DCMRs based on the same criterion in the GWBS data of breast cells. We found that 27% of DCMRs overlapped with DMRs and accounted for 75% of DMR, in which 88% regions had the same change directions in average methylation and CM levels. For the regions with opposite change directions between DMRs and DCMRs, the overlaps of hypo-DMRs and H-DCMRs were greater than the overlaps of hyper-DMRs and L-DCMRs (38 *vs.* 9). The remaining 25% of DMRs were not DCMRs, and two-thirds showed a reduction of average methylation levels in cancer. Although the difference of CM fraction in the remaining 25% of DMRs was not more than 0.2, two-thirds of regions showed a slightly higher CM fraction. The phenomenon that hypo-DMRs exhibited a higher CM fraction suggested that adjacent CpGs might prefer concordant methylation in tumors.

Although CellMethy infers CMRs based on BS-seq data, it is different from some DMR detection tools such as BSmooth^25^. DMR detection tools usually identify DMRs between two types of samples, e.g., normal and cancer, through a comparison of average methylation level. A majority of DMRs may bury the differential methylation pattern. However, the opposite is not always true. There are a large number of regions with different methylation patterns showing similar average methylation levels among different samples that reflect different epigenetic regulatory mechanisms. We focus on the region that shows a concordant methylation pattern of all CpGs and quantify the extent of concordant methylation in a single sample. MethylPurify is a statistical algorithm that uses sequencing reads showing discordant methylation levels to infer tumor purity from tumor samples^26^. This algorithm focuses on the heterogeneity between tumorous and normal cells and infers tumor purity from tumor samples based on the assumption that tumor tissues often contain normal cells. Sequencing data from a tissue are frequently heterogeneous due to being composed of various cells. We focused on the heterogeneity of methylation patterns in both tumor and normal cells, further identifying the regions or markers that can reflect the proportion of tumor cells showing a specific methylation pattern.

Each cell population, especially in a tumor, may contain multiple cell subpopulations, which could have tremendous therapeutic implications. There are existing clinical therapies that may target the most prevalent cells but do not complement all cellular sub-types contained within the population, so the tumors always come back. To optimize therapy, differential drugs and operation methods should be adopted according to the composition of tumor cells. Human cancers harbor epigenetic alterations, such as DNA methylation, that can be dynamically altered. Moreover, some regions of the promoter have shown methylation heterogeneity within individual metastatic tumors^27^. The heterogeneity of DNA methylation may contribute to the heterogeneity of cells from the same cell type. Landan *et al.* found that regional DNA methylation patterns within a cell population from the same cell type were highly polymorphic, both in normal and tumorous cells^21^. They observed reduced levels of epipolymorphism in testicular and H1 ESC populations, which were dominated by completely methylated or unmethylated patterns compared with other somatic cells. We obtained similar results in testicular and H1 ESC populations, which revealed increased levels of CM compared with other somatic cells. In addition, Landan *et al.* found that the epipolymorphism of cancer was lower than normal control samples in hypermethylated regions but similar in hypomethylated regions. Although the distribution of methylation patterns was not further explored in hypomethylated regions, the frequency of concordantly methylated pattern is increased in hypermethylated regions with an average methylation level of 60–70%. The results were partial agreement with our observation that higher differences were observed between cancer and normal cells in the moderately methylated regions (0.2 ~ 0.8).

There are many DNA methylation patterns within a cell population, and we did not infer the fractions of all methylation patterns in a cell population. A concordantly methylated pattern of adjacent CpGs was selected because local hyper-methylation is one of the primary features of the cancer epigenome. Although we only focused on the concordantly methylated pattern, CellMethy can be applied to other methylation patterns to further explore the constituents of cells. This method may further understanding of the dynamic changes in DNA methylation patterns during the development and differentiation of cells, and potentially target a specific cell subpopulation to support personalized cancer therapy.

## Methods

### Data and processing

Three datasets of DNA methylation were downloaded from the Encode (http://genome.ucsc.edu/ENCODE/) and SRA databases (http://www.ncbi.nlm.nih.gov/sra/). The Encode datasets included DNA methylation data on 11 normal and 12 cancer samples through the RRBS technique, including samples of embryonic stem cells (H1 ESC), skin fibroblasts (BJ), mammary epithelial cells (HMEC), skeletal muscle cells (Hsmm), B-lymphocytes (Gm12891, Gm12892), pancreas, skeleton, skin, testis, uterus, lung cancer (A549), colon cancer (Hct), endometrial carcinoma (Ecc1), neuroblastoma (Be2c), acute megakaryocytic leukemia cells (Cmk), cervical carcinoma (Helas), hepatocellular carcinoma (Hepg2), promyelocytic leukemia cells (Hl60), T cell leukemia (Jurkat), leukemia (K562), prostate cancer (Lncap), and breast cancer (Mcf). GWBS data were downloaded from the SRA database (accession no. SRP006728), including HCC and HMEC as a control. A human reference genome was downloaded from Ensemble (HG19). All short sequence fragments from the three datasets were aligned to the human reference genome through *bismark* respectively. If there were multiple replicates in one tissue, all sequence fragments were merged, and the DNA methylation status of CpGs from each read was determined.

### CellMethy algorithm

To identify and quantify concordant methylation regions using single-base resolution DNA methylation data, every read resulting from the DNA methylation data was regarded as representative of a methylation state or epiallele. All reads mapping to a CpG represented a mixture of methylation patterns in a cell population. The method began with sliding windows: the window size was defined from 2 to 10 CpGs, and the sliding step was one CpG. Common reads that covered all CpGs in a window were first identified, suggesting that the distance of adjacent CpGs in the window was no more than the length of bisulfite sequence fragment. That is to say, the distance of adjacent CpGs in the window was no more than 100 bp because the length of reads from RRBS data was usually ~100 bp. If the number of common reads was more than 10, we calculated the fraction of reads (*f*) that showed methylation for all CpGs in a window from common reads. Scanning the genome from 5′ to 3′, the *f* value of each window was obtained from each sample in the three datasets. The window containing the highest *f* value in the neighborhood was considered the hot point. We extended the hot spot to both sides of the window and computed the integration of the *f* value (*I*) as follows, until either *dx* or *dy* was greater than 100 bp or the *f* value equaled 0:


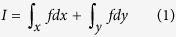


If the region after extension is from *a* to *b* (Fig. 1), then *I* satisfies the following equation:


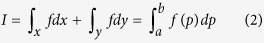


Assuming the maximum value of *f* is *M*, and the minimum value is *m* in the interval [a, b], then


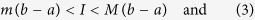



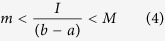


With the assumption that *f*(*p*) is a continuous function on [a, b], the value between *m* and *M* can be reached, i.e., ξ exists that satisfies


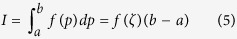


The mean area covered by *f* on the interval [a, b] is equal to the area of a rectangle with edge lengths of (*b − a*) and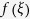. Thus, 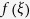 is considered the average size of a cell subpopulation showing full methylation, and is defined as the CM fraction


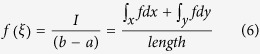


### Simulation data

To determine the appropriate window size, we simulated two datasets that included both a random methylation pattern and a concordant methylation pattern based on genome position and the DNA methylation levels of all CpGs from RRBS data (Bj). A flowchart of the simulation is shown in Supplementary Figure 6. Four different coverage depths (10-, 20-, 50- and 100-fold) were simulated, and the read length was 100 bp. The genome was scanned from 5′ to 3′, and the initial CpG (CpG_0_) and its methylation level were determined. If the distance between CpG_i_ (i = 0, 1, 2, ……) and CpG_i+1_ was greater than 100 bp, CpG_i+1_ was considered a new initial CpG. All reads covering each CpG were allocated to two sets, RS and RC. RS includes the reads that do not cover the next CpG, and RC includes the reads that are shared with the next CpG. The methylation state was determined as 0 or 1, representing unmethylated or methylated, respectively.

Beginning from the initial CpG_0_ site, the relative position of each read was randomly generated and ranged from 1 to 100. The methylation state of CpG_0_ was simulated according to the methylation level. Meanwhile, RC and RS were determined by the relative position of CpG_0_ and the distance between CpG_0_ and CpG_1_. Then, the methylation state of CpG_1_ on each read from RC was simulated. For random methylation simulation data, the methylation state of each read in RC was randomly generated based on the methylation level of CpG_1_. For concordant methylation simulation data, the methylation state of each read in RC was the same as for CpG_0_. Reads in RC and RS were updated according to the distance between CpG_1_ and CpG_2_. In a similar manner, all CpGs in the genome were simulated. If the number of total reads of RC and RS in CpG_i_ was less than the defined coverage depth, new reads were generated and allocated to RC or RS according to the relative position of CpG_i_ and the distance between CpG_i_ and CpG_i+1_. When the relative position of CpG_i_ in the new read minus the distance between CpG_i_ and CpG_j_ (j = i − 1, i − 2, …, 0) was greater than or equal to zero, the methylation state of CpG_j_ was simulated through the previous rule.

### Accuracy evaluation of CellMethy

To evaluate the performance of CellMethy, we simulated 1000 random and concordant methylation regions as negative and positive sets, respectively, with 50-fold coverage depth. The number of CpGs in each region was randomly selected (>5). To control the purity of the negative and positive sets, the methylation level of each region was randomly selected from 0.1 to 0.9. For each region, we simulated negative data through a random methylation pattern and positive data through a concordant methylation pattern (the same as above). In the positive set, the predefined methylation level of each region was considered the true level of CM. In addition, the average methylation levels of CMRs were estimated. AUC values were used to measure the performance of the algorithm.

### Genome region distribution

The position of genes and CpG islands from the human reference genome were downloaded from UCSC (HG19). The promoter was defined as 2 kb upstream from the transcription start site of each gene. Regions with 2 kb distance from the CGI boundary were considered the CGS. The exon, intron, 5′-UTR, 3′-UTR, promoter, and CGS were extracted using *Python*. For each genomic region, the occupancy rate was calculated from the total length of all CMRs located within the region divided by the total length of the corresponding genomic region.

### Identification of differential region

The criteria for differential regions including DMRs and DCMRs referenced the standard of Landan *et al.* which required differences of at least 0.2^21^. Therefore, if the region in the cancer sample showed an increase or decrease in average methylation of at least 0.2 relative to the matched normal sample, the region was regarded as hyper- or hyper-DMR. Similarly, a H-DCMR or L-DCMR was defined as a region with at least a 0.2 increase or decrease in CM level relative to the matched normal sample.

### Availability

CellMethy is open source and available at https://pypi.python.org/pypi/CellMethy/1.1.27.

## Additional Information

**How to cite this article**: Wang, F. *et al.* CellMethy: Identification of a focal concordantly methylated pattern of CpGs revealed wide differences between normal and cancer tissues. *Sci. Rep.* **5**, 18037; doi: 10.1038/srep18037 (2015).

## Supplementary Material

Supplementary Information

## Figures and Tables

**Table 1:** Identification of CMRs based on RRBS data from Encode.

	**Sample**	**NO.**	**Length ± SD**	**NO. CG ± SD**	**CM ± SD**	**M ± SD**
Normal	H1 ESC	2903	39.6 ± 41.03	6 ± 3	0.65 ± 0.31	0.93 ± 0.29
	SF	3533	37.47 ± 38.56	6 ± 2	0.43 ± 0.34	0.78 ± 0.34
	HMEC	3316	35.56 ± 33.35	6 ± 2	0.49 ± 0.36	0.70 ± 0.33
	SMC	10144	38.09 ± 37.96	6 ± 2	0.19 ± 0.30	0.30 ± 0.36
	BL 1	3141	35.74 ± 33.25	6 ± 2	0.52 ± 0.34	0.89 ± 0.27
	BL 2	2431	34.85 ± 30.59	6 ± 2	0.43 ± 0.32	0.71 ± 0.28
	Pancreas	2790	33.39 ± 30.19	6 ± 2	0.34 ± 0.34	0.51 ± 0.36
	Skeleton	3646	36.36 ± 33.75	6 ± 2	0.39 ± 0.35	0.65 ± 0.35
	Skin	4702	37.23 ± 37.42	6 ± 3	0.37 ± 0.35	0.52 ± 0.37
	Testis	3908	36.99 ± 35.79	6 ± 3	0.34 ± 0.33	0.43 ± 0.36
	Uterus	4351	35.88 ± 34.44	6 ± 2	0.37 ± 0.35	0.56 ± 0.37
Normal Mean	4078	36.47	6	0.41	0.63	
Cancer	Lung	5347	45.74 ± 52.10	7 ± 3	0.65 ± 0.33	0.76 ± 0.27
	Colon	7137	49.85 ± 59.29	7 ± 4	0.64 ± 0.35	0.75 ± 0.36
	Endometrium	5019	43.64 ± 49.29	7 ± 3	0.57 ± 0.33	0.77 ± 0.26
	Neuroblastoma	5598	45.31 ± 54.19	7 ± 4	0.51 ± 0.35	0.69 ± 0.32
	AML	6637	50.35 ± 58.80	7 ± 4	0.62 ± 0.34	0.77 ± 0.30
	Cervial	7544	58.87 ± 71.41	8 ± 5	0.69 ± 0.29	0.83 ± 0.24
	Liver	6345	50.27 ± 61.54	7 ± 4	0.52 ± 0.33	0.73 ± 0.27
	PML	5674	38.12 ± 39.70	6 ± 3	0.41 ± 0.37	0.61 ± 0.35
	ACL	4886	39.79 ± 41.56	7 ± 3	0.51 ± 0.35	0.71 ± 0.31
	CLL	5156	51.66 ± 62.36	7 ± 4	0.46 ± 0.31	0.70 ± 0.26
	Prostate	6541	48.21 ± 57.90	7 ± 4	0.48 ± 0.33	0.70 ± 0.29
	Breast	11183	64.08 ± 68.72	8 ± 5	0.63 ± 0.31	0.79 ± 0.28
Cancer Mean	6422	48.82	7	0.56	0.73	

NO.: The number of CMRs whose CM fraction were more than 0, identified from the sample. Length: average length of CMRs corresponding to the sample. SD: standard deviation. NO.CG: average number of CGs in CMRs. CM: average CM fraction of all CMRs corresponding to the sample. M: average methylation level of each sample.

## References

[b1] Wu S. C. & Zhang Y. Active DNA demethylation: many roads lead to Rome. Nat Rev Mol Cell Biol. 11, 607–620 (2010). 20683471 10.1038/nrm2950 PMC3711520

[b2] Smallwood S. A. *et al.* Dynamic CpG island methylation landscape in oocytes and preimplantation embryos. Nat Genet. 43, 811–814 (2011). 21706000 10.1038/ng.864 PMC3146050

[b3] Feng S., Jacobsen S. E. & Reik W. Epigenetic reprogramming in plant and animal development. Science 330, 622–627 (2010). 21030646 10.1126/science.1190614 PMC2989926

[b4] Bird A. DNA methylation patterns and epigenetic memory. Genes Dev. 16, 6–21 (2002). 11782440 10.1101/gad.947102

[b5] Hodges E. *et al.* Directional DNA methylation changes and complex intermediate states accompany lineage specificity in the adult hematopoietic compartment. Mol Cell. 44, 17–28 (2011). 21924933 10.1016/j.molcel.2011.08.026 PMC3412369

[b6] Ji H. *et al.* Comprehensive methylome map of lineage commitment from haematopoietic progenitors. Nature 467, 338–342 (2010). 20720541 10.1038/nature09367 PMC2956609

[b7] Gabel H. W. & Greenberg M. E. Genetics. The maturing brain methylome. Science 341, 626–627 (2013). 23929975 10.1126/science.1242671 PMC4445635

[b8] Lande-Diner L. *et al.* Role of DNA methylation in stable gene repression. J Biol Chem. 282, 12194–12200 (2007). 17311920 10.1074/jbc.M607838200

[b9] Rakyan V. K. *et al.* An integrated resource for genome-wide identification and analysis of human tissue-specific differentially methylated regions (tDMRs). Genome research 18, 1518–1529 (2008). 18577705 10.1101/gr.077479.108 PMC2527707

[b10] Irizarry R. A. *et al.* The human colon cancer methylome shows similar hypo- and hypermethylation at conserved tissue-specific CpG island shores. Nat Genet. 41, 178–186 (2009). 19151715 10.1038/ng.298 PMC2729128

[b11] Meissner A. *et al.* Genome-scale DNA methylation maps of pluripotent and differentiated cells. Nature 454, 766–770 (2008). 18600261 10.1038/nature07107 PMC2896277

[b12] Noushmehr H. *et al.* Identification of a CpG island methylator phenotype that defines a distinct subgroup of glioma. Cancer Cell. 17, 510–522 (2010). 20399149 10.1016/j.ccr.2010.03.017 PMC2872684

[b13] Hansen K. D. *et al.* Increased methylation variation in epigenetic domains across cancer types. Nat Genet. 43, 768–775 (2011). 21706001 10.1038/ng.865 PMC3145050

[b14] Berman B. P. *et al.* Regions of focal DNA hypermethylation and long-range hypomethylation in colorectal cancer coincide with nuclear lamina-associated domains. Nat Genet. 44, 40–46 (2012). 22120008 10.1038/ng.969 PMC4309644

[b15] Timp W. & Feinberg A. P. Cancer as a dysregulated epigenome allowing cellular growth advantage at the expense of the host. Nat Rev Cancer. 13, 497–510 (2013). 23760024 10.1038/nrc3486 PMC4636434

[b16] Broske A. M. *et al.* DNA methylation protects hematopoietic stem cell multipotency from myeloerythroid restriction. Nat Genet. 41, 1207–1215 (2009). 19801979 10.1038/ng.463

[b17] Trowbridge J. J., Snow J. W., Kim J. & Orkin S. H. DNA methyltransferase 1 is essential for and uniquely regulates hematopoietic stem and progenitor cells. Cell Stem Cell. 5, 442–449 (2009). 19796624 10.1016/j.stem.2009.08.016 PMC2767228

[b18] Baylin S. B. & Jones P. A. A decade of exploring the cancer epigenome - biological and translational implications. Nat Rev Cancer. 11, 726–734 (2011). 21941284 10.1038/nrc3130 PMC3307543

[b19] Luu P. L., Scholer H. R. & Arauzo-Bravo M. J. Disclosing the crosstalk among DNA methylation, transcription factors, and histone marks in human pluripotent cells through discovery of DNA methylation motifs. Genome Res. 23, 2013–2029 (2013). 24149073 10.1101/gr.155960.113 PMC3847772

[b20] Suzuki M. M. & Bird A. DNA methylation landscapes: provocative insights from epigenomics. Nat Rev Genet. 9, 465–476 (2008). 18463664 10.1038/nrg2341

[b21] Landan G. *et al.* Epigenetic polymorphism and the stochastic formation of differentially methylated regions in normal and cancerous tissues. Nat Genet. 44, 1207–1214 (2012). 23064413 10.1038/ng.2442

[b22] Eckhardt F. *et al.* DNA methylation profiling of human chromosomes 6, 20 and 22. Nat Genet. 38, 1378–1385 (2006). 17072317 10.1038/ng1909 PMC3082778

[b23] Ladd-Acosta C., Aryee M. J., Ordway J. M. & Feinberg A. P. Comprehensive high-throughput arrays for relative methylation (CHARM). Curr Protoc Hum Genet. Chapter 20, Unit 20 21 21–19 (2010). 20373514 10.1002/0471142905.hg2001s65 PMC6353553

[b24] Ghasemali S. *et al.* Inhibitory effects of beta-cyclodextrin-helenalin complexes on H-TERT gene expression in the T47D breast cancer cell line - results of real time quantitative PCR. Asian Pac J Cancer Prev. 14, 6949–6953 (2013). 24377631 10.7314/apjcp.2013.14.11.6949

[b25] Hansen K. D., Langmead B. & Irizarry R. A. BSmooth: from whole genome bisulfite sequencing reads to differentially methylated regions. Genome Biol. 13, R83 (2012). 23034175 10.1186/gb-2012-13-10-r83 PMC3491411

[b26] Zheng X. *et al.* MethylPurify: tumor purity deconvolution and differential methylation detection from single tumor DNA methylomes. Genome Biol. 15, 419 (2014). 25103624 10.1186/s13059-014-0419-x PMC4165374

[b27] Aryee M. J. *et al.* DNA methylation alterations exhibit intraindividual stability and interindividual heterogeneity in prostate cancer metastases. Sci Transl Med. 5, 169ra110 (2013). 10.1126/scitranslmed.3005211 PMC3577373 23345608

