# Supplementary material for: CellMethy: Identification of a focal concordantly methylated pattern of CpGs revealed wide differences between normal and cancer tissues
Source: Sci Rep. 2015 Dec 10;5:18037. doi: 10.1038/srep18037 (PMC4674876; doi:10.1038/srep18037)
Supplement: Supplementary Information [file srep18037-s1.pdf]

CellMethy: Identification of a focal concordantly methylated pattern of CpGs revealed wide differences between normal and cancer tissues

Fang Wang[1†], Shaojun Zhang[1†], Hongbo Liu[1], Yanjun Wei[1], Yihan Wang[1], Xiaole Han[1], Jianzhong Su[1], Dongwei Zhang[2], Baodong Xie[3*], Yan Zhang[1*]

1. College of Bioinformatics Science and Technology, Harbin Medical University, Harbin, 150081
2. The 2nd Affiliated Hospital, Harbin Medical University, Harbin, 150081, China
3. The Department of Cardiovascular Surgery, The Second Affiliated Hospital, Harbin Medical University, Harbin, 150081, China

*Corresponding author: Yan Zhang, College of Bioinformatics Science and Technology, Harbin Medical University, 194 Xuefu Road, Harbin, 150081, China. E-mail: tyozhang@ems.hrbmu.edu.cn; baodongxie_doctor@aliyun.com

† Equal contributors.

Supplementary data

Supplementary Figure 1. Evaluation of CMRs number between random and concordant methylation pattern. Length of windows from 2 to 10 CpGs (A) - (I). Red represents concordant methylation simulation data with different coverage, and black represents random methylation data.

Supplementary Figure 2. Evaluation of cumulative probability distribution of CG number in CMRs between random and concordant methylation pattern. Length of windows from 2 to 10 CpGs (A) - (I). Red represents concordant methylation simulation data with different coverage, and black represents random methylation data.

Supplementary Figure 3. Evaluation of probability density distribution of CM fraction between random and concordant methylation pattern. Length of windows from 2 to 10 CpGs (A) - (I). Red represents concordant methylation simulation data with different coverage, and black represents random methylation data.

Supplementary Figure 4. Relationship between average methylation levels and CM fractions in each cancer. Each point is a CMR, the black line is the fitted curve of the cancer and gray is the average fitted curve of all normal tissues/cells.

Supplementary Figure 5. Differences in CM fraction between breast cancer and normal cell line from genome wide BS-Seq data. (A) Box plot of CM fractions and average methylation levels between breast cancer and normal. * represents p value of Wilcoxon test less than 0.01. (B) Differential degree of CM fractions and average methylation between breast cancer and normal in eight genome regions.

Supplementary Figure 6. Flowchart of simulation data.

Supplementary Table 1. Identification of CMRs based on genome wide BS-Seq data of breast cancer.

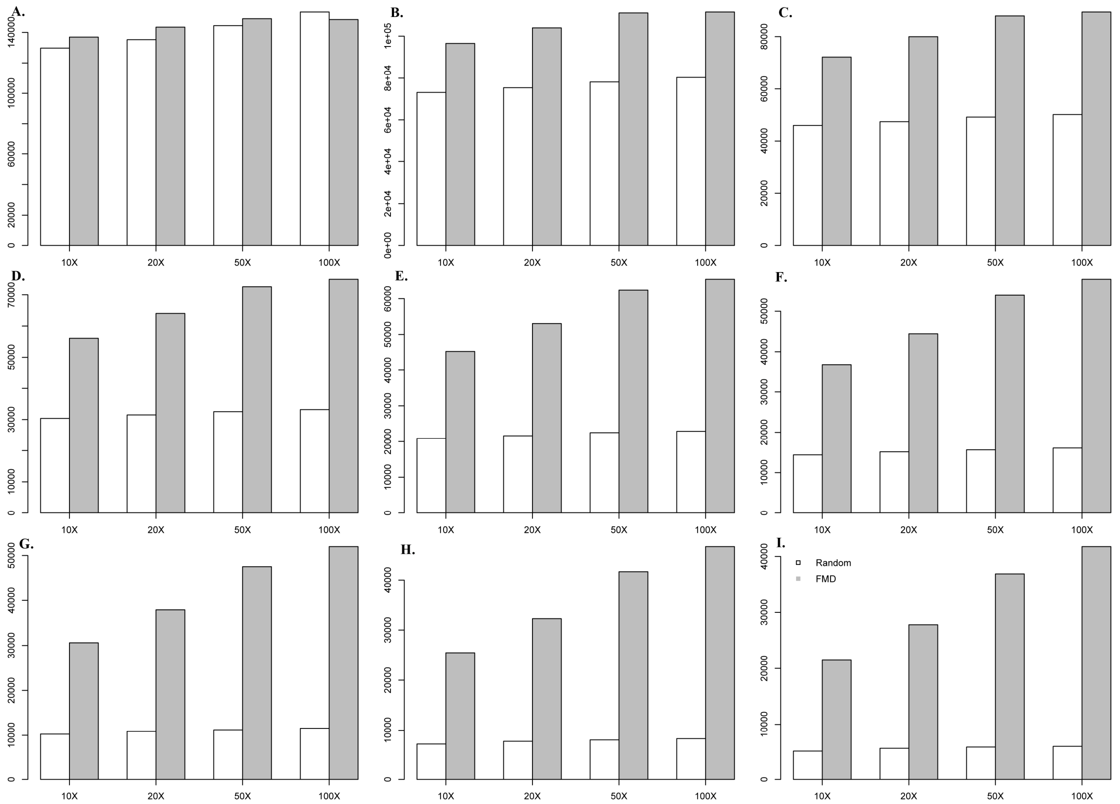

Supplementary Figure 1. Evaluation of CMRs number between random and concordant methylation pattern. Length of windows from 2 to 10 CpGs (A) - (I). Red represents concordant methylation simulation data with different coverage, and black represents random methylation data.

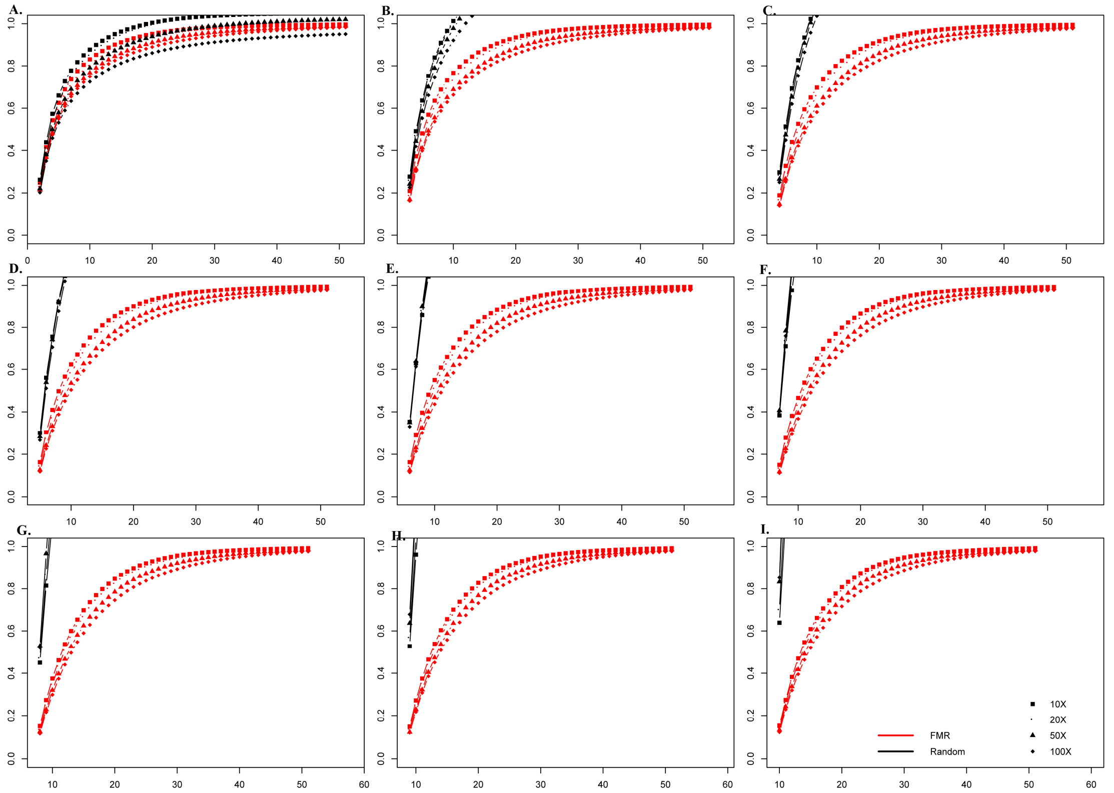

Supplementary Figure 2. Evaluation of cumulative probability distribution of CG number in CMRs between random and concordant methylation pattern. Length of windows from 2 to 10 CpGs (A) - (I). Red represents concordant methylation simulation data with different coverage, and black represents random methylation data.

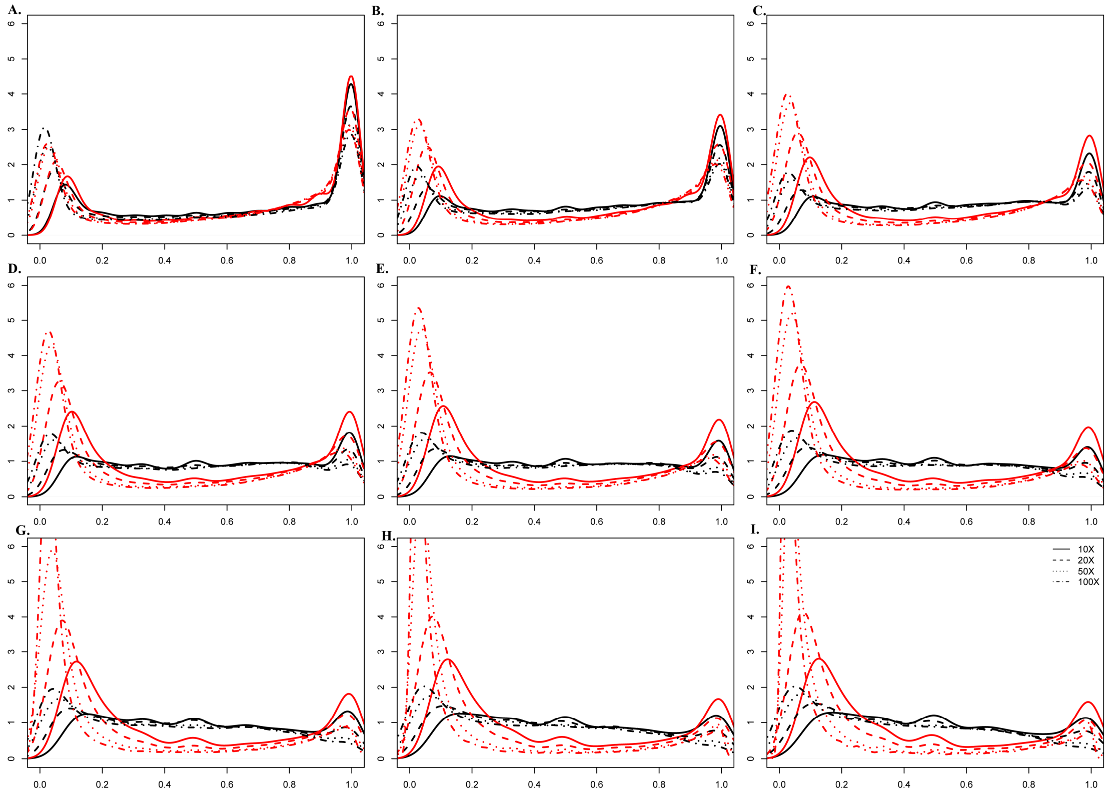

Supplementary Figure 3. Evaluation of probability density distribution of CM fraction between random and concordant methylation pattern. Length of windows from 2 to 10 CpGs (A) - (I). Red represents concordant methylation simulation data with different coverage, and black represents random methylation data.

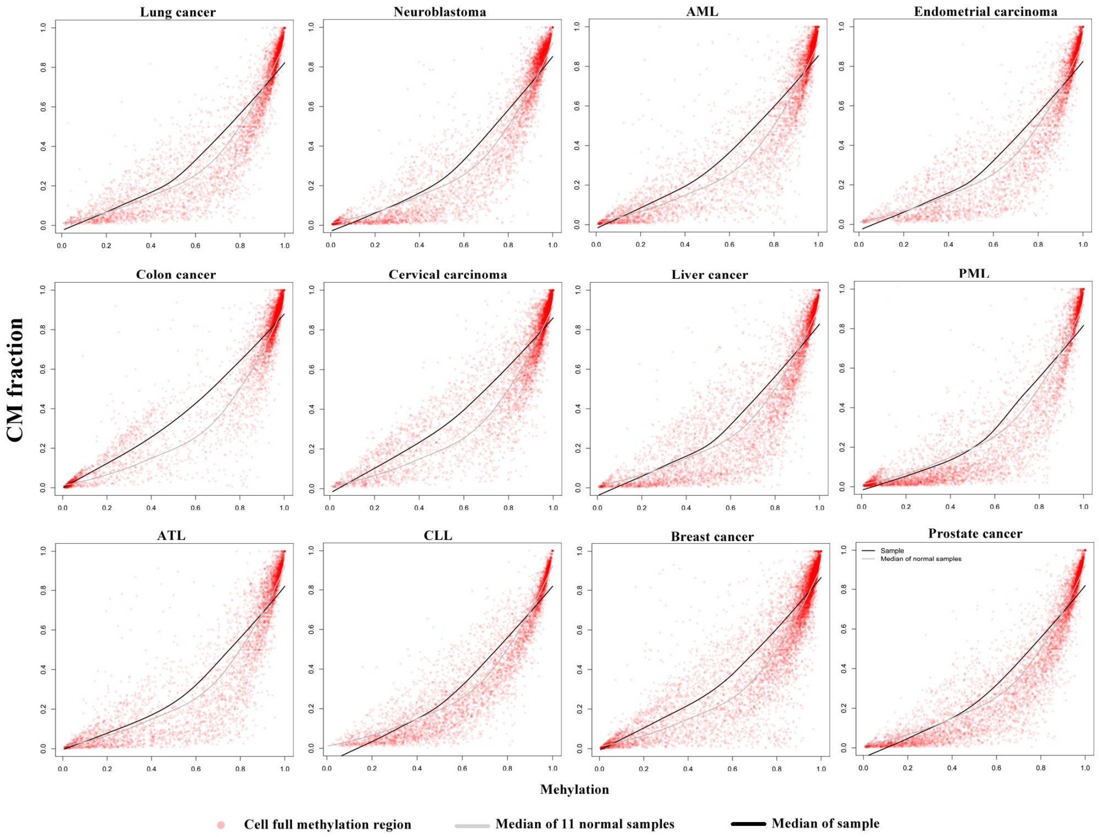

Supplementary Figure 4. Relationship between average methylation levels and CM fractions in each cancer. Each point is a CMR, the black line is the fitted curve of the cancer and gray is the average fitted curve of all normal tissues/cells.

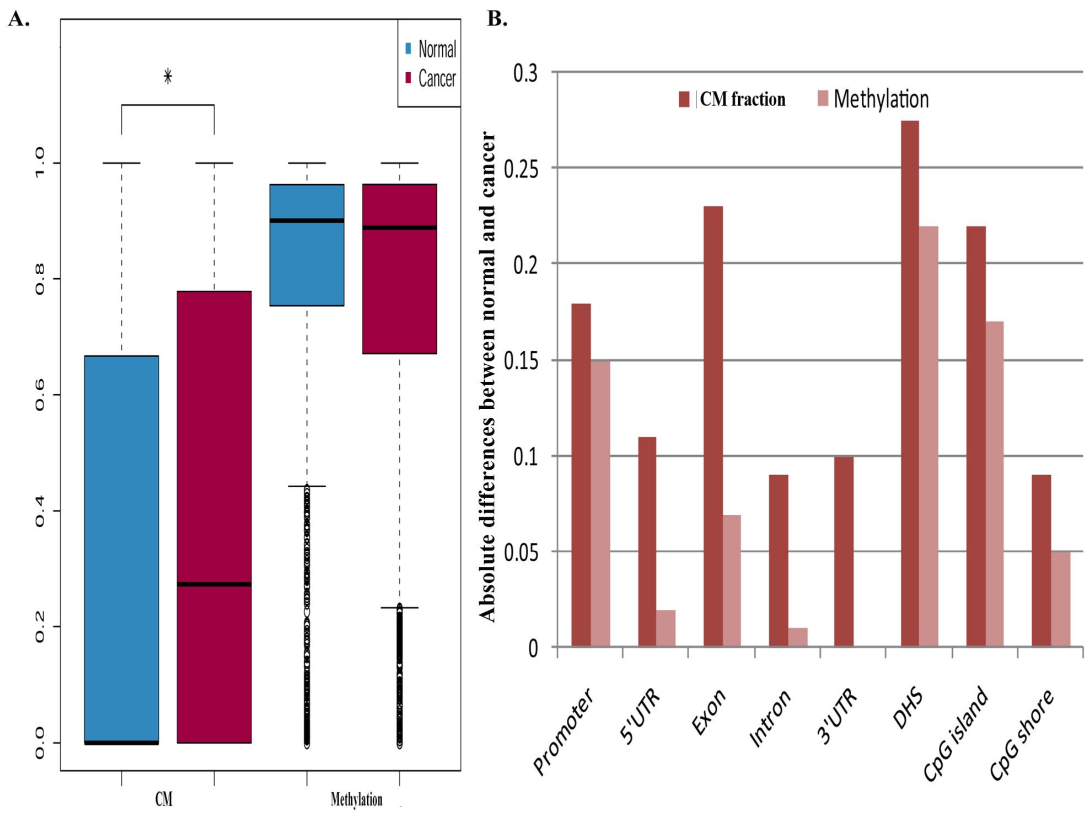

Supplementary Figure 5. Differences in CM fraction between breast cancer and normal cell line from genome wide BS-Seq data. (A) Box plot of CM fractions and average methylation levels between breast cancer and normal. * represents p value of Wilcoxon test less than 0.01. (B) Differential degree of CM fractions and average methylation between breast cancer and normal in eight genome regions.

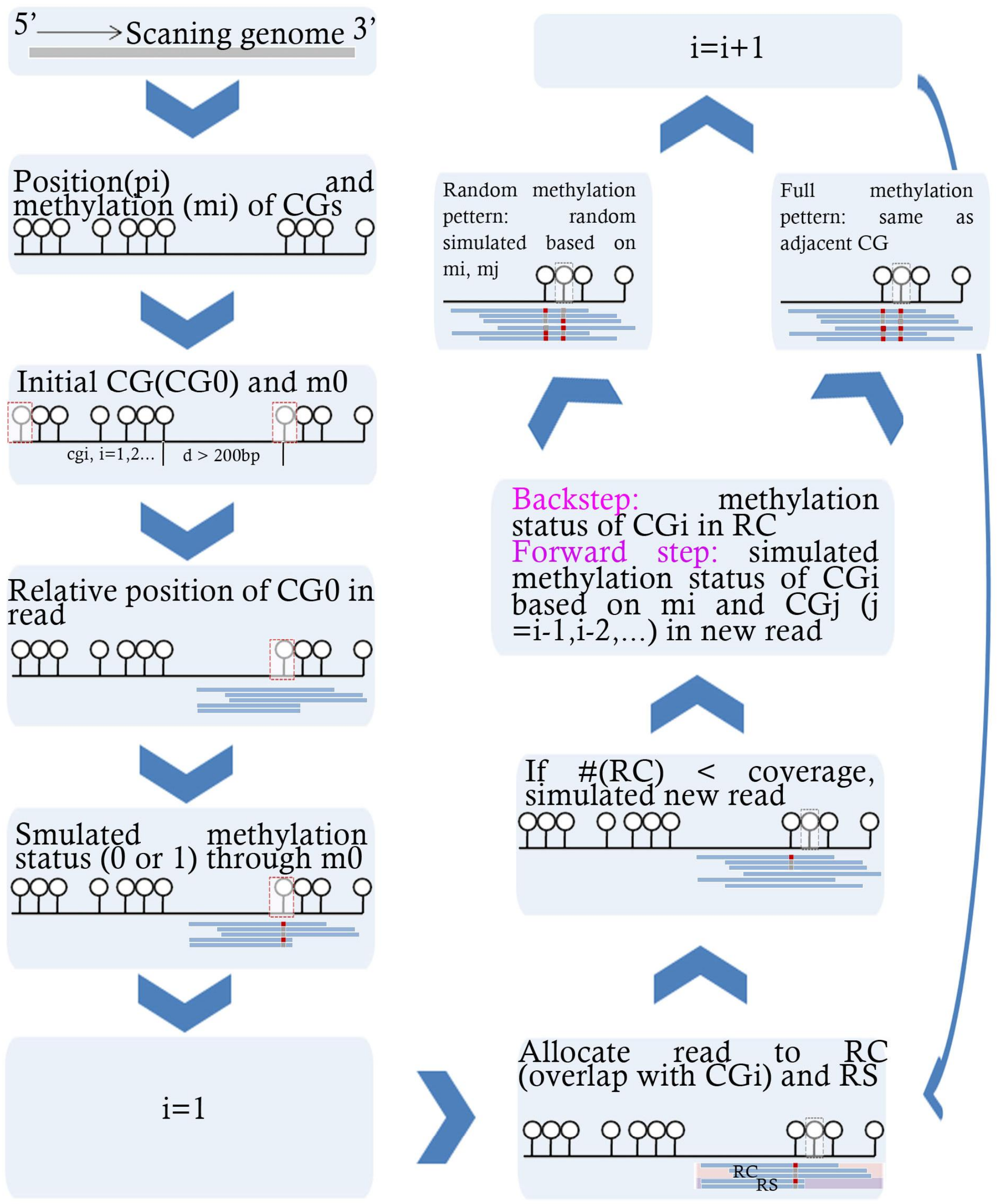

Supplementary Figure 6. Flowchart of simulation data.

Supplementary Table 1. Identification of CMRs based on genome wide BS-Seq data of breast cancer

Sample	NO	CG ± SD	length ± SD	FMC ± SD	Meth ± SD
Normal	835	5 ± 1	50.82 ± 29.18	0.60 ± 0.28	0.91 ± 0.16
Cancer	1093	6 ± 2	55.4 ± 35.55	0.67 ± 0.30	0.90 ± 0.21
